# 

**Published:** 1865

**Authors:** 


### OF THE WEST.

EDITED AND PUBLISHED BY J. TAFT.

MARCH & APRIL, 1865.

MONTHLY:
*Three Dollars per Annum, in Advance.*

CINCINNATI:
WRIGHTSON & CO., Printers, 167 WALNUT STREET.

*J. TAFT, Dentist, 172 Race Street.*

